# Supplementary material for: Constitutive inflammation and epithelial-mesenchymal transition dictate sensitivity to nivolumab in CONFIRM: a placebo-controlled, randomised phase III trial
Source: Nat Commun. 2025 Jul 21;16:6688. doi: 10.1038/s41467-025-61691-4 (PMC12280041; doi:10.1038/s41467-025-61691-4)
Supplement: Supplementary file 2 — Reporting Summary [file 41467_2025_61691_MOESM2_ESM.pdf]

nature portfolio

Corresponding author(s): Professor Dean A. Fennell

Last updated by author(s): Nov 18, 2024

Reporting Summary

Nature Portfolio wishes to improve the reproducibility of the work that we publish. This form provides structure for consistency and transparency in reporting. For further information on Nature Portfolio policies, see our Editorial Policies and the Editorial Policy Checklist.

Statistics

For all statistical analyses, confirm that the following items are present in the figure legend, table legend, main text, or Methods section.

n/a	Confirmed	
☐	☒	The exact sample size (*n*) for each experimental group/condition, given as a discrete number and unit of measurement
☐	☒	A statement on whether measurements were taken from distinct samples or whether the same sample was measured repeatedly
☐	☒	The statistical test(s) used AND whether they are one- or two-sided<br>*Only common tests should be described solely by name; describe more complex techniques in the Methods section.*
☒	☐	A description of all covariates tested
☒	☐	A description of any assumptions or corrections, such as tests of normality and adjustment for multiple comparisons
☐	☒	A full description of the statistical parameters including central tendency (e.g. means) or other basic estimates (e.g. regression coefficient) AND variation (e.g. standard deviation) or associated estimates of uncertainty (e.g. confidence intervals)
☐	☒	For null hypothesis testing, the test statistic (e.g. *F*, *t*, *r*) with confidence intervals, effect sizes, degrees of freedom and *P* value noted<br>*Give P values as exact values whenever suitable.*
☒	☐	For Bayesian analysis, information on the choice of priors and Markov chain Monte Carlo settings
☒	☐	For hierarchical and complex designs, identification of the appropriate level for tests and full reporting of outcomes
☐	☒	Estimates of effect sizes (e.g. Cohen's *d*, Pearson's *r*), indicating how they were calculated

Our web collection on statistics for biologists contains articles on many of the points above.

Software and code

Policy information about availability of computer code

Data collection

Provide a description of all commercial, open source and custom code used to collect the data in this study, specifying the version used OR state that no software was used.

Data analysis

All statistical analyses were done with Stata (version 16.0).
Whole exome sequencing: Clean reads FASTP
Sequence Alignment: Burrows-Wheeler Aligner (bwa-0.7.17)
Mapped genomes sorting: Sambamba (v0.6.7)
Duplicate reads: Picard tools
SNV and INDEL calling: VarScan2 somatic v2.3 and MuTect2 in the GATK bundle (4.0.5.1)
somatic copy number alternations (SCNA): ASCAT
HRD scores: scarHRD R package
HLA typing: Polysolver(v1.0)
HLA loss of heterozygosity: LOHHLA
Neoantigen prediction: NetMHC and NetMHCpan
RNA sequencing:
Removal of low quality reads:Fastp (0.12.2)
Quality assessment of RNA sequencing: RSeQC (v5.0.3)
Processed read alignment: STAR (2.6.1d)
Gene assignment of reads:HTSeq
Count normalisation (FPKM): Deseq2
Fusion detection: Arriba (v.2.1.0), STAR-Fusion (1.9.0)

Immune repertoire analysis: TRUST4 (v1.0.0)
Immune deconvolution: quantiseq, EPIC, MCP counter CIBERSORTx
Geneset enrichment analysis: fGSEA R package, ggplot2 R package
ERV expression: ERVmap, ht-seq, DEseq
Digital image analysis: Python (version 3.6, package pandas 1.1.5), R (version 4.2.3), inForm 2.6.0 image analysis software (Akoya Biosciences, Marlborough, MA, USA)
Graphing software: Prism 9.5.1 (Graphpad,San Diego, CA, USA)

For manuscripts utilizing custom algorithms or software that are central to the research but not yet described in published literature, software must be made available to editors and reviewers. We strongly encourage code deposition in a community repository (e.g. GitHub). See the Nature Portfolio guidelines for submitting code & software for further information.

Data

Policy information about availability of data

All manuscripts must include a data availability statement. This statement should provide the following information, where applicable:

- Accession codes, unique identifiers, or web links for publicly available datasets
- A description of any restrictions on data availability
- For clinical datasets or third party data, please ensure that the statement adheres to our policy

Data availability

Trial data relating to this publication shall remain confidential to the sponsor organisation and will not be disclosed, except when disclosure might be required in accordance with pharmacovigilance duties of the parties involved. Individual participant data can be made available, after deidentification, to investigators who provide a written request in accordance with General Data Protection Regulation and following authorisation from the sponsor organisation, starting immediately and ending 3 years after publication. Data sharing requests should be directed to D.A.F and G.O.G. Southampton Clinical Trials Unit (SCTU), University of Southampton, Southampton, UK, is committed to the responsible sharing of clinical trial data and trial samples with the wider research community. Data access is administered through the SCTU Data Release Committee. Requests for data access and sharing for SCTU trials should be emailed to the SCTU Data Release Committee Coordinator at ctu@soton.ac.uk.

The WES and RNA-sequencing raw data is available in SRA Run Selector. The data can be publicly accessed upon publication via

https://www.ncbi.nlm.nih.gov/bioproject/PRJNA1148791, which is hosted by the National Centre for Biotechnology Information, under accession number PRJNA916814. All of the other data supporting the findings of this study are available within the article and its supplementary information files and from the corresponding author upon reasonable request. Source data are provided with this paper.

Research involving human participants, their data, or biological material

Policy information about studies with human participants or human data. See also policy information about sex, gender (identity/presentation), and sexual orientation and race, ethnicity and racism.

Reporting on sex and gender	Data is only provided for sex which was collected data in this study
Reporting on race, ethnicity, or other socially relevant groupings	not applicable
Population characteristics	CONFIRM was a multicentre, double-blind, placebo-controlled, parallel group, randomised phase 3 trial design. Enrolment involved 24 hospitals in the UK. Patients were eligible if they were aged 18 years or over with histologically confirmed pleural or peritoneal mesothelioma of any histological subtype, and who had been previously treated with at least one course of standard of care, chemotherapy with radiologically confirmed progression. Patients required an Eastern Cooperative Oncology Group (ECOG) performance status score of 0 or 1, radiologically assessable disease according to modified Response Evaluation Criteria in Solid Tumors (RECIST) or RECIST version 1.1, and an archival tumour biopsy for blinded multi-omic analysis.<br>The following laboratory criteria were mandated: a white blood cell count of at least 2 × 109 cells per L, neutrophil count at least 1·5 × 109 cells per L, platelet count at least 100 × 109 per L, haemoglobin concentration at least 90 g/L, serum creatinine concentration of up to 1·5 × the upper limit of normal (ULN) or creatinine clearance higher than 50 mL/min (using the Cockcroft-Gault formula). Liver function tests i.e., aspartate aminotransferase concentration was allowed up to 3 × ULN or alanine aminotransferase concentration up to 3 × ULN (if both are assessed, both needed to be up to 3 × ULN), and total bilirubin concentration up to 1·5 × ULN (except patients with Gilbert syndrome, who had to have total bilirubin <51.3 µmol/L).<br>Patients were approached in the hospital setting by research staff. There was no restriction on the number of previous therapies received. Key exclusion criteria included previous treatment with an immune checkpoint inhibitor, uncontrolled metastasis involving the CNS, and autoimmune disease. The complete eligibility criteria are provided in the study protocol (supplementary materials). Median survival with no additional treatment was expected to be approximately 6 months for eligible patients.<br>The study protocol was approved by the West Midlands, Edgbaston Research Ethics Committee (16/WM/0472). Further analysis of formalin fixed tissue acquired at the time of routine clinical care (extended pleurectomy decortication) was conducted under research ethics approval 14/LO/1527, a translational research platform entitled Predicting Drug and Radiation Sensitivity in Thoracic Cancers. The study was approved by the University Hospitals of Leicester NHS Trust (reference IRAS131283 and 14/EM/1159) with the University of Leicester being a sponsor. The study was completed in accordance with the provisions of the Declaration of Helsinki and Good Clinical Practice guidelines as defined by the

	International Conference on Harmonisation. Written informed consent was obtained from all patients before enrolment.
Recruitment	patients with the above key inclusion criteria
Ethics oversight	Sponsor - The University of Southampton. The study protocol was approved by the West Midlands, Edgbaston Research Ethics Committee (16/WM/0472). Further analysis of formalin fixed tissue acquired at the time of routine clinical care (extended pleurectomy decortication) was conducted under research ethics approval 14/LO/1527, a translational research platform entitled Predicting Drug and Radiation Sensitivity in Thoracic Cancers. The study was approved by the University Hospitals of Leicester NHS Trust (reference IRAS131283 and 14/EM/1159) with the University of Leicester being a sponsor. The study was completed in accordance with the provisions of the Declaration of Helsinki and Good Clinical Practice guidelines as defined by the International Conference on Harmonisation. Written informed consent was obtained from all patients before enrolment.

Note that full information on the approval of the study protocol must also be provided in the manuscript.

Field-specific reporting

Please select the one below that is the best fit for your research. If you are not sure, read the appropriate sections before making your selection.

☒ Life sciences ☐ Behavioural & social sciences ☐ Ecological, evolutionary & environmental sciences

For a reference copy of the document with all sections, see nature.com/documents/nr-reporting-summary-flat.pdf

Life sciences study design

All studies must disclose on these points even when the disclosure is negative.

Sample size	N=332
Data exclusions	not applicable -no data was excluded
Replication	not applicable - patient level data and drug response is unique to the individual
Randomization	221 patients were randomised to receive nivolumab or placebo (111 patients) all of whom were included in the analysis
Blinding	double-blind randomised phase III clinical trial

Reporting for specific materials, systems and methods

We require information from authors about some types of materials, experimental systems and methods used in many studies. Here, indicate whether each material, system or method listed is relevant to your study. If you are not sure if a list item applies to your research, read the appropriate section before selecting a response.

Materials & experimental systems

n/a	Involved in the study	
☐	☒	Antibodies
☒	☐	Eukaryotic cell lines
☒	☐	Palaeontology and archaeology
☒	☐	Animals and other organisms
☐	☒	Clinical data
☒	☐	Dual use research of concern
☒	☐	Plants

Methods

n/a	Involved in the study	
☒	☐	ChIP-seq
☒	☐	Flow cytometry
☒	☐	MRI-based neuroimaging

Antibodies

Antibodies used	CD8 C8/144B 1:200 Dako (Target:"T-cell surface glycoprotein CD8 alpha chain (Human); UniProt P01732 - CD8A_HUMAN"; clone:C8/144B; host: mouse; clonality: monoclonal; conjugates: unconjugated; specificity: human; manufacturer or supplier: Dako/ Agilent; manufacturer website link: https://www.agilent.com/en/product/immunohistochemistry/antibodies-controls/primary-antibodies/cd8-(concentrate)-76631, citations: 691 citations listed in CiteAb records); CD4 4B12 1:50 Dako (Target: T-cell surface glycoprotein CD4 (Human); UniProt: P01730 - CD4_HUMAN clone 4B12; host: mouse; clonality: monoclonal; conjugates: unconjugated; specificity: human; manufacturer website link: concentrate discontinued, only version for Autostainer link 48 available, citations: 150 citations listed in CiteAb records); PD1 EH33 1:200 Cell Signalling Technology (Target: "Programmed cell death protein 1 (Human); UniProt: Q15116 - PDCD1_HUMAN"; clone: EH33; host: Mouse; clonality: monoclonal; conjugates: unconjugated; specificity: human; manufacturer website link: https://www.cellsignal.com/product/productDetail.jsp?productId=43248&utm_medium=b2b&utm_campaign=general, citations: 27 citations listed in CiteAb records); CD19 EPR5906

	1:300 Abcam (Target: B-lymphocyte antigen CD19 (Human); UniProt: P15391 - CD19_HUMAN; clone: EPR5906; host: rabbit; clonality: recombinant monoclonal; conjugates: unconjugated; specificity: human; manufacturer website link: https://www.abcam.com/products/primary-antibodies/cd19-antibody-epr5906-ab134114.html, citations: 74 citations listed in CiteAb records); TIM3 D5D5R 1:200 Cell Signalling Technology (Target: Hepatitis A virus cellular receptor 2 (Human); UniProt: Q8TDQ0 - HAVR2_HUMAN; clone: D5D5R; host: rabbit; clonality: recombinant monoclonal; conjugates: unconjugated; specificity: human; manufacturer website link: https://www.cellsignal.com/products/primary-antibodies/tim-3-d5d5r-xp-rabbit-mab/45208, citations: 51 citations listed in CiteAb records); TIGIT E5Y1W 1:50 Cell Signalling Technology (Target: T-cell immunoreceptor with Ig and ITIM domains (Human); UniProt: Q495A1 - TIGIT_HUMAN; clone: E5Y1W; host: rabbit; clonality: recombinant monoclonal; conjugates: unconjugated; specificity: human; manufacturer website link: https://www.cellsignal.com/products/primary-antibodies/tigit-e5y1w-xp-rabbit-mab/99567, citations: 11 citations listed in CiteAb records)
Validation	All antibodies were acquired commercially and had been validated by the manufacturer as specified in the information sheet All antibodies were acquired commercially and had been validated by the manufacturer as specified in the information sheet. Further experimental quality control was applied by assessment for specific staining by a senior histopathology biomedical scientist

Clinical data

Policy information about clinical studies

All manuscripts should comply with the ICMJE guidelines for publication of clinical research and a completed CONSORT checklist must be included with all submissions.

Clinical trial registration	NCT03063450
Study protocol	Confirm Version 8 12 Mar 2021
Data collection	10th May 2017 to 30th March 2020, data was collected at each trial site
Outcomes	The co-primary endpoints were investigator reported PFS as the time from randomisation to disease progression according to blinded investigator assessment or death, whichever occurred first, and OS i.e., the time from randomisation to death from any cause. Secondary endpoints were overall response to treatment, defined as either complete or partial response according to masked investigator assessment, stable disease, or progressive disease; 12-month OS and PFS; safety; and efficacy (for PFS and OS) according to tumour PD-L1 tumour proportion score.

Plants

Seed stocks	not applicable
Novel plant genotypes	not applicable
Authentication	not applicable
